# Pneumocephalus After Anterior Lumbar Spinal Surgery Due to Trauma: A Case Report

**DOI:** 10.7759/cureus.37726

**Published:** 2023-04-17

**Authors:** Tomoyuki Takigawa, Takuya Morita, Takuya Taoka, Takeshi Ishihara, Yasuo Ito

**Affiliations:** 1 Department of Orthopaedic Surgery, Kobe Red Cross Hospital, Kobe, JPN

**Keywords:** lumbar burst fracture, cerebrospinal fluid leakage, dural injury, anterior lumbar spinal surgery, pneumocephalus

## Abstract

Pneumocephalus as a complication of anterior lumbar spinal surgery is extremely rare. A 53-year-old male patient presented with L4 fracture. Posterior fixation from L3 to L5 was conducted one day after the trauma. As the patient’s neurological deficit persisted, additional anterior surgery by L4 vertebral body replacement was performed on the 19th day. Both surgeries were completed without obvious intraoperative complications. Two weeks after the anterior lumbar surgery, the patient complained of severe headaches, and computed tomography scan revealed pneumocephalus and massive fluid retention in the abdomen. The symptoms improved with conservative treatment, including bed rest, spinal drainage, intravenous drip infusion, and prophylactic administration of antibiotics. Due to the lack of tamponade effect in the soft tissues, a large amount of cerebrospinal fluid leakage may induce and cause progression of pneumocephalus in anterior dural injury.

## Introduction

The first reported case of cranial pneumocephalus was from an autopsy of a trauma patient [1]. Pneumocephalus can be induced by a variety of causes, including head trauma and intracranial surgery. It has been reported that 74% of pneumocephalus is associated with cranial trauma [2]. Pneumocephalus occurring as a complication of spinal surgery is extremely rare. A literature review conducted in 2021 found only 25 cases of pneumocephalus associated with spinal surgery [3]. Since then, two more papers have been published in 2023 [4,5]. The majority, 18 of the 27 cases, were in the lumbar spine, and the others were in the cervical, thoracic, and thoracolumbar spine, all in three cases. Posterior decompression (14/27) and fixation (8/27) were the main surgical procedures. Our literature search revealed no reports of pneumocephalus after anterior lumbar surgery. This report aims to discuss one of the rare complications of anterior lumbar spine surgery, pneumocephalus, and to highlight the lessons learned from this case.

## Case presentation

**Figure 1:**
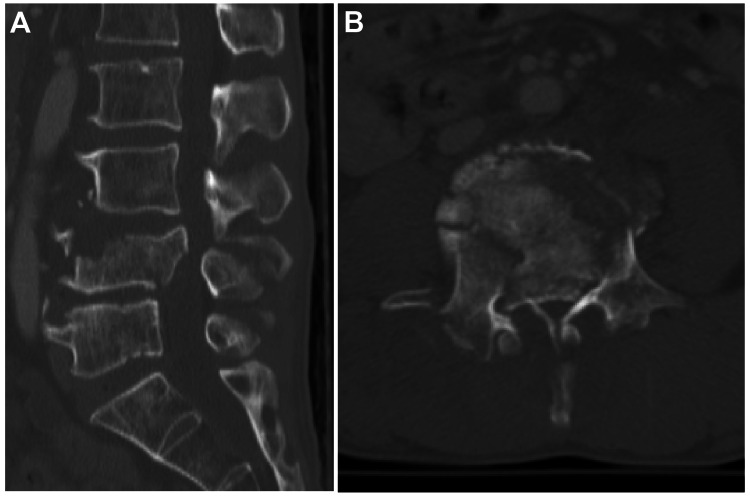
Preoperative CT scan (A: sagittal and B: axial) showed a complete burst fracture of L4. A fractured bone fragment protruded into the spinal canal.

A 53-year-old male patient with no past medical history and no regular medications was injured by a fall from a fourth-floor balcony to the ground after drinking. Contrast-enhanced computed tomography (CT) scan of the entire body, including the head and the entire spine, revealed an L4 burst fracture, bilateral lung contusions, hemopneumothorax, and mandibular fractures (Figures 1A, 1B).

**Figure 2:**
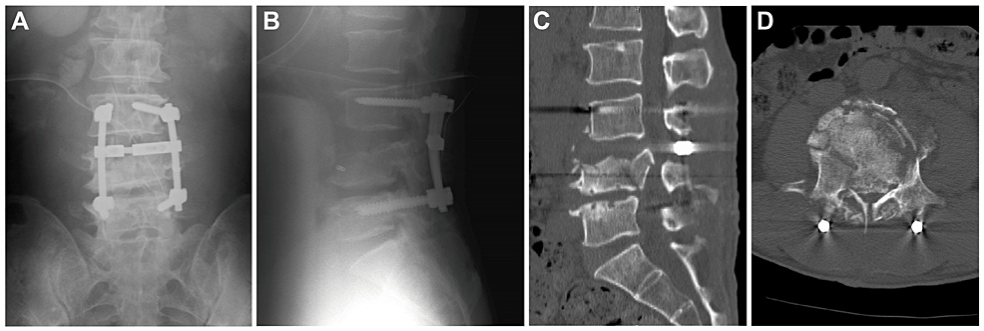
Radiographs (A: anteroposterior and B: lateral) and CT scan (C: sagittal and D: axial) after posterior fixation from L3 to L5. A bony protrusion into the spinal canal persisted.

**Figure 3:**
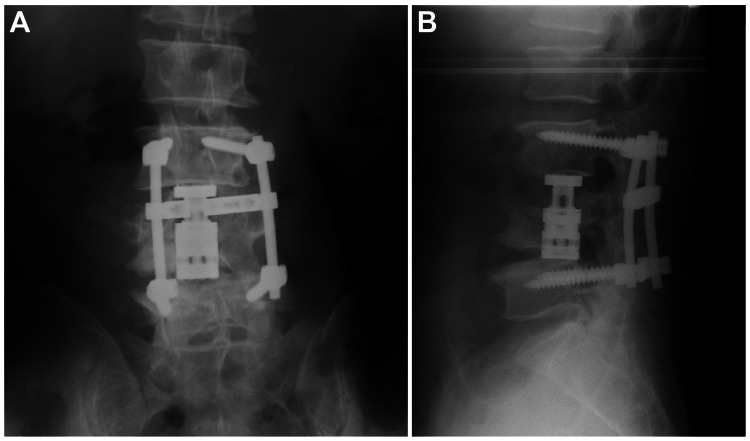
Radiographs (A: anteroposterior and B: lateral) after posterior and anterior fixation from L3 to L5.

The patient had sensory deficits below both thighs and incomplete lower limb paralysis of Frankel C. One day after the trauma, posterior open fixation using pedicle screws from L3 to L5 without decompression was applied (Figures 2A-2D). The patient’s neurological deficit did not improve and severe stenosis at L4 remained after the posterior surgery. To remove the bone fragments in the spinal canal and replace L4 vertebral body with a cage, additional anterior surgery was performed on the 19th day of the injury (Figures 3A, 3B).

**Figure 4:**
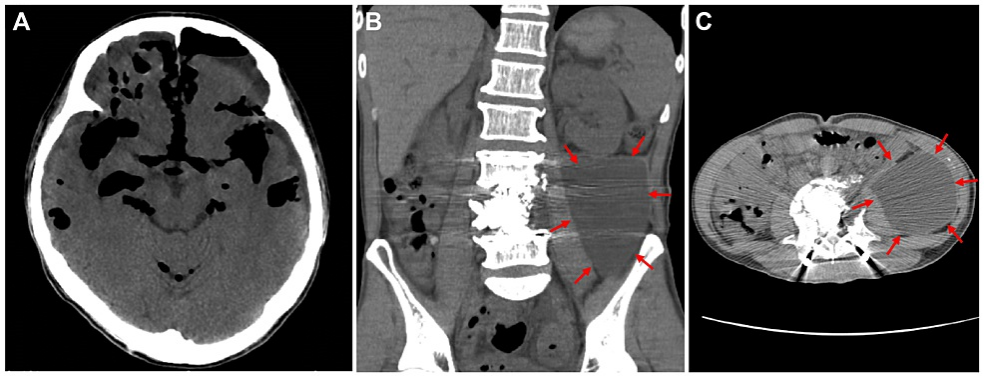
A CT scan shows a large amount of air in the cranium (A) and massive fluid retention in the abdomen (arrows, B: coronal and C: axial).

Both surgeries were completed without complications. However, two weeks after the anterior lumbar surgery, the patient complained of severe headaches. A CT scan showed a large amount of air in the cranium and massive fluid retention in the abdomen (Figures 4A-4C).

**Figure 5:**
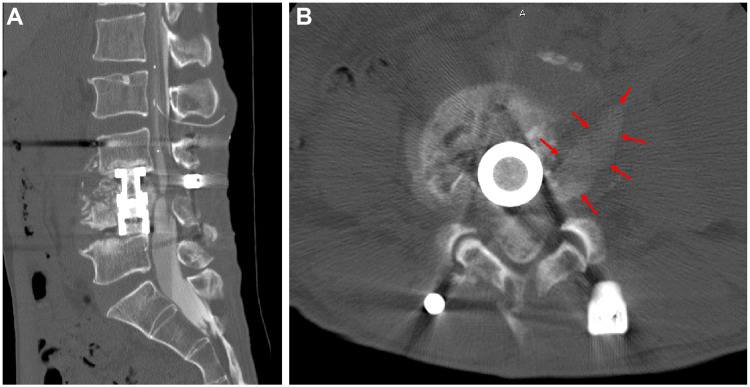
A postmyelography CT scan in sagittal (A) and axial (B) planes revealed leakage of contrast medium at the L4 level into the retroperitoneal space (arrows).

A postmyelography CT scan revealed leakage of contrast medium at the L4 level into the retroperitoneal space, and a diagnosis of pneumocephalus was made, due to inapparent dural injury and spinal fluid leakage (Figures 5A, 5B).

**Figure 6:**
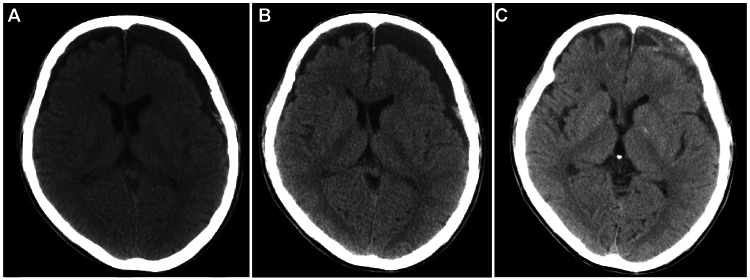
Head CT scan at two weeks (A), three weeks (B), and six weeks (C) after conservative treatment. Air in the cranium disappeared at two weeks, resulting in subdural hygroma, which then developed into a chronic subdural hematoma (C).

The patient was placed on bed rest. A spinal drainage tube from L2/3 to L1/2 level was placed in the patient, and prophylactic antimicrobials and continuous intravenous drip infusion were administered for one week. The symptoms disappeared and imaging improved within two weeks. The patient was released from bed rest and started walking rehabilitation at three weeks. He had no symptoms of headache at six weeks, although the head CT scan showed a change from subdural hygroma to a chronic subdural hematoma (Figure 6A-6C). His urinary dysfunction remained due to a neurogenic bladder and required intermittent self-catheterization. The patient could walk with a single cane and was transferred to a rehabilitation hospital.

## Discussion

Most pneumocephalus is associated with head trauma, and it is very rarely associated with spinal trauma or spinal surgery [3-11]. Hentschel et al. reported two cases of pneumocephalus related to thoracic tumor resection surgery and cerebrospinal fluid (CSF) leak [6]. Sasaki et al. reported pneumocephalus associated with CSF fistula as a complication of cervical spinal surgery [7]. Nowak et al. reported intracranial subdural hematoma and pneumocephalus related to a misplaced pedicle screw in spinal scoliosis surgery [8]. Most of these cases were related to intraoperative dural injury and CSF leaks. Pneumocephalus associated with late presenting CSF leak after uneventful lumbar surgery has also been reported [4,9,10]. An intraoperative dural tear or CSF leak was not obvious in the current case. However, the course of events would indicate dural and arachnoid injury, either at the time of the initial trauma, during the anterior surgery, or postoperatively. The development of the dural injury at the initial trauma is unlikely because a dural injury would have been detected during the surgeries or by imaging. Since the metal cage was placed close to the dura mater, the possibility that postoperative rubbing of the dura mater against the cage caused the dural injury cannot be completely ruled out.

CSF leakage is commonly identified in cases of dural injury due to the positive CSF pressure (7-20 cm H_2_O); however, if the CSF pressure drops after a CSF leak and equilibrates at atmospheric pressure, the CSF leak may not be evident even in cases of dural injury. CSF leaks in posterior spinal surgery often become apparent in the form of a fistula through the surgical approach. However, in anterior lumbar spine surgery, the spinal fluid leak does not become apparent on the body surface due to the large retroperitoneal space between the surgical incision and the site of the dural injury. In the lumbar anterior surgery, large amounts of CSF leakage may occur because there is no tamponade effect in the soft tissues. From a diagnostic standpoint, a postmyelography CT scan was helpful to identify a persistent CSF leak in the abdomen.

Several mechanisms have been proposed to explain how pneumocephalus is induced after spinal surgery, such as the following: rapid air movement in the dural canal (inverted pop bottle model); check valving at the site of dural injury (ball valve model); negative pressure in the dural canal (negative pressure model); and elastic changes in the CSF compartment (elastic model) [12]. In the present case, a combination of these mechanisms occurred, and a large amount of CSF leakage was an aggravating factor. The CSF leak and air influx mechanism can be explained using a closed plastic bottle (Videos 1, 2).

**Video 1:** When surface tension and atmospheric pressure are in equilibrium, water does not flow out from the plastic bottle, even if there is a hole. When the plastic bottle is squeezed, water leaks and air enters instead.

**Video 2:** When a plastic bottle is cut wide open, water does not flow out if surface tension and atmospheric pressure are balanced. However, when a pressure difference is created by tilting the surface of the water, water starts to spill out and a large amount of air enters instead.

We speculate that the CSF pressure changes and intradural inflow of air were caused by the patient’s body movement, coughing, etc., followed by the rapid movement of air into the cranium and rapid onset of the disease.

Due to its rarity, there is no guideline for the treatment of pneumocephalus associated with spinal surgery; however, it has been reported that most cases of pneumocephalus improve with conservative treatment, including bed rest, intravenous drip infusion, and prophylactic administration of antibiotics [13]. Nowak et al. reported emergent craniotomy and subdural drainage followed by the second surgical dural repair [8]. In the current case, myelography and postmyelography CT scan confirmed the diagnosis and exact location of the dural injury, and a drainage tube was placed in the lumbar subarachnoid space to control the amount of spinal fluid leaking into the abdominal cavity. The patient's symptoms and pneumocephalus improved with conservative treatment; however, surgical treatment would have been necessary had the patient's symptoms not improved. In the previous reports, surgical treatment was required in six of 27 cases [3-5].

The pneumocephalus eventually became a chronic subdural hematoma. We believe that pneumocephalus created a void, which was initially filled with subdural hygroma and gradually mixed with blood components to form a subdural hematoma. No special treatment was given to this patient because his symptoms did not deteriorate and there were no findings of compression of the brain parenchyma on imaging. Long-term follow-up is required, and surgical attention may be necessary when symptoms such as cognitive function or paralysis develop.

## Conclusions

The occurrence of pneumocephalus as a complication of spinal surgery is extremely rare. This report aimed to discuss and highlight lessons learned from a case of pneumocephalus as a complication of anterior lumbar spine surgery. We conclude that a large amount of CSF leakage may occur after spinal surgery and may induce pneumocephalus, even if an intraoperative dural tear is not obvious. Spinal surgeons should be aware that pneumocephalus can occur after anterior lumbar surgery for spinal trauma.
